# No association between resistance mutations, empiric antibiotic, and mortality in ceftriaxone-resistant *Escherichia coli* and *Klebsiella pneumoniae* bacteremia

**DOI:** 10.1038/s41598-018-31081-6

**Published:** 2018-08-24

**Authors:** Shi Thong Heng, Swaine L. Chen, Joshua G. X. Wong, David C. Lye, Tat Ming Ng

**Affiliations:** 1 Department of Pharmacy, Tan Tock Seng Hospital, Singapore, Singapore; 2 GERMS and Infectious Diseases Group, Genome Institute of Singapore, Singapore, Singapore; 3 Department of Medicine, Yong Loo Lin School of Medicine, National University of Singapore, Singapore, Singapore; 4 Department of Infectious Diseases, Tan Tock Seng Hospital, Singapore, Singapore; 5 Lee Kong Chian School of Medicine, Nanyang Technological University, Singapore, Singapore

## Abstract

The objective of this study was to correlate resistance mutations of extended spectrum beta-lactamases (ESBL) and AmpC beta-lactamases and virulence factors (VF) with 30-day mortality in patients treated with either piperacillin-tazobactam or carbapenems. A post-hoc analysis on 123 patients with ceftriaxone-resistant *Escherichia coli* and *Klebsiella pneumoniae* bacteremia treated empirically with piperacillin-tazobactam and carbapenems was performed. Beta-lactamase resistance mutations and VF were identified by whole genome sequencing (WGS). The primary endpoint was 30-day mortality. Multivariate analyses were performed using logistic regression. WGS showed diverse multilocus sequence types (MLST) in 43 *K. pneumoniae* strains, while ST131 predominated in *E. coli* strains (57/80). CTX-M was most commonly detected (76/80 [95%] of *E. coli*; 39/43 [91%] of *K pneumoniae*.), followed by OXA (53/80 [66%] of *E. coli*; 34/43 [79%] of *K. pneumoniae*). A significant correlation was found between the number of genes encoding third-generation cephalosporin-resistant beta-lactamases and 30-day mortality (p = 0.045). The positive association was not significant after controlling for empiric carbapenem, Pitt score 3 and *K. pneumoniae* (OR 2.43, P = 0.073). None of the VF was associated with 30-day mortality. No association was found between 30-day mortality and any ESBL and AmpC beta-lactamases or VF when piperacillin-tazobactam or carbapenems were given. No significant association between 30-day mortality and active empiric therapy was found.

## Introduction

Extended-spectrum and AmpC beta-lactamase *Enterobacteriaceae*, which lead to resistance to third generation cephalosporins, have become a global problem^1–3^. ESBL enzymes have been well studied since 1980’s, with hundreds of variants reported, including the TEM, SHV, and CTX-M families^4–6^. Multiple genetic mechanisms including plasmid transmission have facilitated the spread of CTX-M beta-lactamases^7^ worldwide, including in Singapore^8^. CTX-M genes have been commonly reported in *Escherichia coli* sequence type 131 (ST131)^9^, which was the leading cause of multidrug-resistant *E. coli* infections in the United States in 2011-2012^10^. The AmpC beta-lactamases are also known to be transmissible among *Enterobacteriaceae* via plasmids^11^, and was previously reported in Singapore^8^. Partially due to their increased antibiotic resistance, infections by ESBL- and AmpC-producing bacteria result in increased mortality^12–15^.

ESBL- and AmpC-producing *Enterobacteriaceae* are susceptible to carbapenems, which have become the drug of choice for treatment of ESBL producing gram negative bacteremia^16,17^. However, the rapid rise of carbapenem-resistant *Enterobacteriaceae* globally^18–20^, partially driven by the increased use of carbapenems, has left little treatment options for common bacterial pathogens such as *E. coli* and *Klebsiella pneumoniae*^21,22^. To mitigate the rise of carbapenem resistance in *Enterobacteriaceae*, exploring possible effective alternative antibiotics to treat ESBL and AmpC beta-lactamase producers as a carbapenem-sparing strategy is a viable therapeutic approach.

The effectiveness of piperacillin-tazobactam against ESBL-producing bacteria has been described. The reduced efficacy in the presence of high bacterial inoculum has been described *in vitro*^23–26^. There are mixed results in clinical studies comparing efficacy of piperacillin-tazobactam and carbapenems^27–31^. An ongoing randomized trial aims to address the question of whether piperacillin-tazobactam is non-inferior to carbapenems for definitive treatment of ESBL gram negative bacteremia^32^. However, concerns of complex co-resistance mechanisms, including enzymes not well inhibited by tazobactam or clavulanate (e.g., plasmid-derived AmpC), may deter clinicians from using piperacillin-tazobactam for ESBL producing gram negative bacteremia^33^. This study aims to correlate resistance mutations and virulence factors of ESBL and AmpC beta-lactamases with 30-day mortality in patients with ceftriaxone-resistant *E. coli* and *K. pneumoniae* bacteremia when treated empirically with active piperacillin-tazobactam and carbapenems.

## Method

### Study setting and population

This analysis was reported according to the STROBE guidelines^34^. A post-hoc analysis was performed on ceftriaxone-resistant *E. coli* and *K. pneumoniae* bacteremic isolates from patients between August 2011 and May 2013 at Tan Tock Seng Hospital. The patients were included in a previously published retrospective cohort study by Ng *et al.*^28^. Briefly, patients with ceftriaxone-resistant *E. coli* and *K. pneumoniae* bacteremia were identified from electronic microbiology databases. For patients with multiple episodes of ceftriaxone-resistant *E. coli* or *K. pneumoniae* bacteremia, only the first episode was included. Patients were excluded if they had polymicrobial bacteremia, or did not receive at least 48 hours of empirical or definitive antimicrobial therapy. Other data collected included patient demographics, antibiotic susceptibility, empiric and definitive antibiotic therapy, source of bacteremia, Charlson’s co-morbidity index, Pitt bacteremia score, and 30-day mortality^28^. Ethics approval for this study was obtained from National Healthcare Group domain specific review boards (Approval number 2013/00083). Patient information was anonymized and de-identified prior to data collection and analysis in accordance with institutional guidelines and review board approvals, which included an approved waiver of informed consent.

In this study, we examined the effect of ESBL and AmpC resistance mutations, and virulence factors on 30-day mortality of patients with *E. coli* and *K. pneumoniae* bacteremia, treated with piperacillin-tazobactam or a carbapenem as active empiric therapy. We included Pitt bacteremia score to adjust for severity of illness.

Beta-lactamase genes that confer third-generation cephalosporin resistance according to the Bush and Jacoby functional classification were identified for further analyses^35,36^. AmpC genes were included as third-generation cephalosporin-resistant variants. Genes of the class SHV-OKP-LEN are closely related in sequence and grouped together in the resistance gene database we used (see below). Only some of these genes are ESBL, however, and not all alleles have been classified. We therefore performed separate analyses considering all SHV-OKP-LEN genes to be either ESBL or non-ESBL.

### DNA extraction and sequencing library preparation

Each isolate from the clinical laboratory was streaked out to single colonies on LB-agar. A single colony was inoculated into Luria-Bertani broth (Gibco) and cultured overnight at 37 °C with agitation overnight. Cells from 1 ml of this culture were collected by centrifugation at 14, 000 g 1 minute. Genomic DNA was isolated from the resulting pelleted bacteria using the QIAamp DNA mini kit (Qiagen). DNA samples were quantified using a QUBIT 2.0 fluorometer (Invitrogen). Sequencing libraries were prepared with the Nextera XT Library Prep Kit (Illumina) according to the manufacturer’s instructions. The adapters were indexed using either the Nextera XT Index Kit (Illumina). Finally, 10 nM of each sample DNA sequencing library was pooled together and sequenced on a HiSeq 4000 (Illumina) with a 2 × 151 run.

### Sequence analysis

Raw FASTQ reads were processed with using standard GERMS (Genome Institute of Singapore (GIS) Efficient and Rapid Microbial Sequencing) platform pipelines. Briefly, both de novo and reference-based analysis were used. De novo assemblies were performed using the Velvet assembler (version 1.2.10) with parameters optimized by Velvet Optimiser (k-mer length ranging from 81 to 127)^37^, scaffolded with Opera (version 1.4.1), and finished with FinIS (version 0.3)^38,39^. Genomes were initially annotated with Prokka (version 1.11)^40^. Resistance genes were called using BLASTN with a minimum identity of 90% over 90% of the gene length required for calling a gene present. MLST calls were made by using a custom BLASTN-based MLST caller.

Reference-based analyses were performed using the *E. coli* ST131 reference strain EC958 genome (GenBank accession NZ HG941719.1)^41,42^ as the reference for *E. coli* strains and *K. pneumoniae* HS11268 (GenBank accession NC 016845.1) as the reference for *K. pneumoniae* strains. FASTQ files were mapped using bwa (version 0.7.10)^43^; indel realignment and SNP (single nucleotide polymorphism) calling was performed using Lofreq* (version 2.1.2) with default parameters^44^.

Phylogenetic trees were made by calculating a dissimilarity matrix using SNPRelate^45^ and inferring a neighbor-joining tree using APE (version 3.5)^46^. All phylogenetic trees were visualized with GGTREE 3.2^47^. All R packages were run in R (3.2.2). Multilocus sequence typing, resistance genes, and virulence factors were called directly from the raw FASTQ files using SRST2 (version 0.1.8)^48^ with default settings using the ARGAnnot database^49^ for resistance genes and the VFDB database^50^ for virulence factors. Resistance genes and MLST calls were cross-validated with the calls from the de novo assemblies, with discrepancies resolved by manual inspection of the raw data.

Novel MLST alleles and sequence types were submitted to Enterobase (for *E. coli*) and the Institut Pasteur MLST/cgMLST databases (for *K. pneumoniae*). All raw sequencing data has been deposited in GenBank under BioProject PRJNA431029.

### Statistical analysis

Categorical variables were compared using the Chi-square or Fisher’s exact tests where applicable while continuous variables were compared using the Mann–Whitney U test between *E. coli* and *K. pneumoniae* bacteremia cases. A P-value of <0.05 was considered significant. The presence of beta-lactamase resistance mutations were also compared between empiric piperacillin-tazobactam and carbapenem groups using Chi-square tests. The number of third-generation cephalosporin-resistant beta-lactamase genes and mortality rate were compared using the linear-by-linear association test of Mantel-Haenszel. To assess the impact of number of resistant genes on patient mortality, a multivariate analysis was performed using binary logistic regression. Empiric carbapenem therapy, *K. pneumoniae* bacteremia as well as clinical (Pitt bacteremia score) and microbiological variables (Number of third-generation cephalosporin resistant genes in each isolate) with p values of <0.1 in univariate analysis were included into the model (Supplementary data); source of infection was omitted from multivariate analysis as the majority of cases (61%) were urinary. All analyses were performed using SPSS version 21 (IBM Corp, Armonk, NY, USA).

## Results

The published clinical study by Ng *et al*. included 151 patients with ceftriaxone-resistant *E. coli* and *K. pneumoniae* bacteremia treated with active empiric monotherapy including either piperacillin-tazobactam or a carbapenem^28^. Only 124 bacteremic isolates were viable when revived from stocks from the microbiology department of our hospital. We included all of these 124 isolates for this study.

### General features of strains

We performed whole genome sequencing on all 124 strains (80 *E. coli*, 44 *K. pneumoniae*). One of these strains was originally identified as an *E. coli* but, when sequenced, was very similar to *K. pneumoniae* reference sequences; this was excluded from further analysis. We obtained a median of 5.4 million paired end reads per strain (range 1.7–8.8 million), representing a coverage (assuming a 5Mbp genome) of 325 (range 102–526).

By multilocus sequence typing (MLST), the *K pneumoniae* strains were very diverse. Eight of 43 strains had novel MLST profiles. Among these 8 novel profiles, the most common profile accounted for only 3 strains. The most common MLST was ST307, accounting for 6 of 43 strains.

In contrast, by MLST the *E. coli* strains were dominated by ST131 (57/80 strains, including one single locus variant). The next most common sequence types, with 4 strains each, were ST38 and ST1193; neither is closely related to ST131.

**Figure 1:**
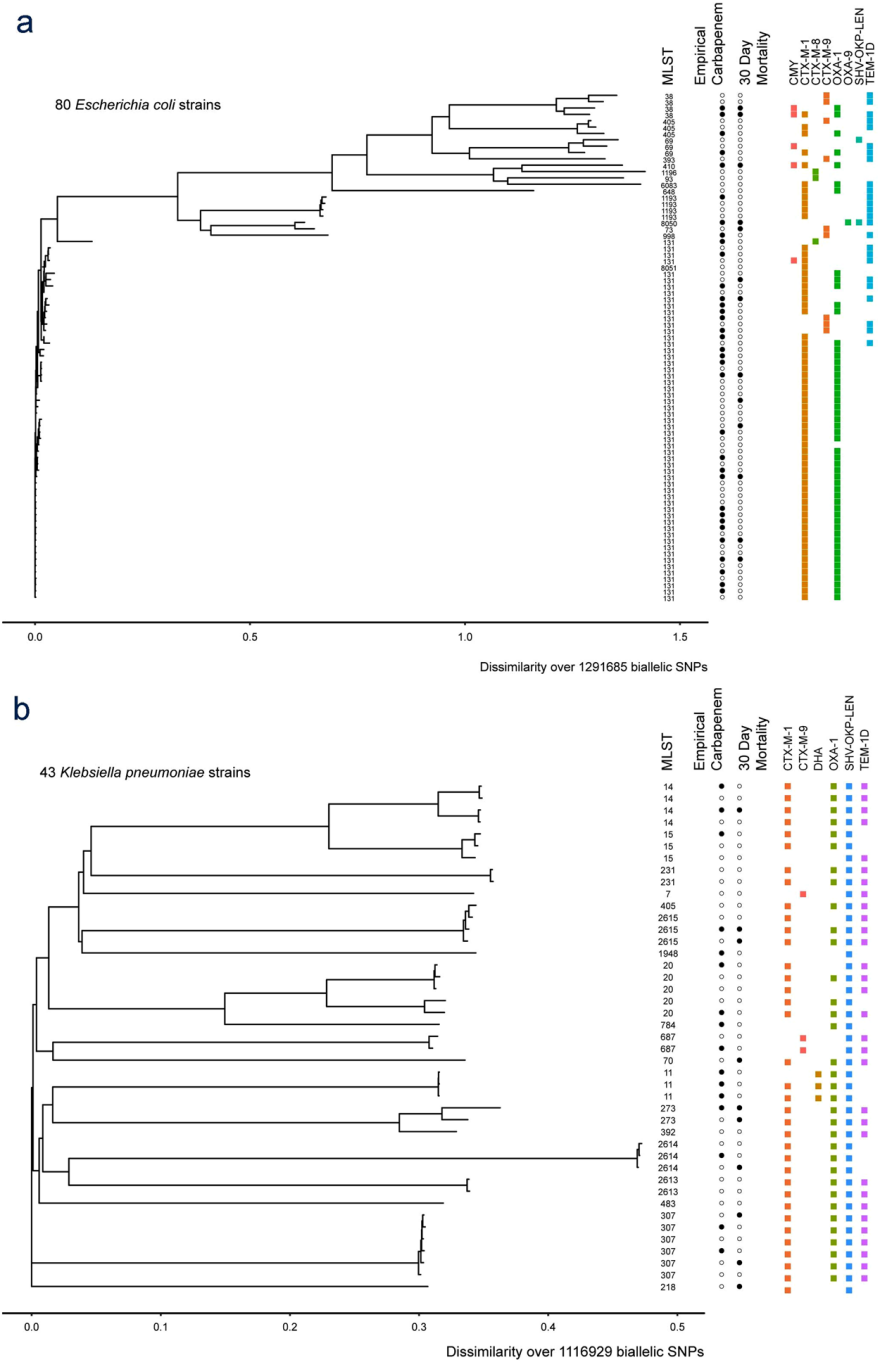
Phylogenetic trees for (**a**) *E. coli* and (**b**) *K. pneumoniae* isolates from this study. Neighbor joining trees were constructed from whole genome SNP profiles based on a reference-based analysis. MLST for each isolate is shown on the right. Black circles indicate yes (filled) or no (open) empirical carbapenem treatment and 30-day mortality, as indicated by the header. On the far right, colored squares indicate presence of a gene encoding the indicated resistance based on sequencing data.

As indicated by the MLST results, the isolates obtained in this study were overall quite diverse with the exception of the ST131 strains. Separate whole genome SNP-based phylogenetic trees of the *K. pneumoniae* and *E. coli* strains are shown in Fig. 1; these demonstrate uniform clustering of related MLST types as well as the diversity of the strains across MLST types overall.

### Beta-lactamase gene analysis

The prevalence of resistance mutations in beta-lactamase genes is shown in Table 1, according to classifications on the publicly available database. The distribution of CTX-M and OXA were similar in both *E. coli* and *K. pneumoniae* groups; TEM-1D and SHV were predominantly found in *K. pneumoniae* samples. The frequency of AmpC was low in this population. Mortality rate and distribution of Pitt bacteremia, Charlson, and APACHE II scores in *E. coli* and *K. pneumoniae* groups were similar. 70% of *K. pneumoniae* strains were categorized as nosocomial compared to 26% with *E. coli* strains. The sources of bacteremia were similar between *E. coli* and *K. pneumoniae* groups (Table 1).

**Table 1:** Beta-lactamases and clinical characteristics of patients with third-generation cephalosporin-resistant *E. coli* and *K. pneumoniae* bacteremia.

	Type of bacteremia		
	*E. coli* (n = 80)	*K. pneumoniae* (n = 43)	p-value
CTX-M	76 (95.0)	39 (90.7)	0.356
CTX-M-1^a^	64 (84.2)	36 (92.3)	0.614
CTX-M-8^a^	3 (3.9)	0 (0)	0.199
CTX-M-9^a^	9 (11.8)	3 (7.7)	0.446
OXA	53 (66.3)	34 (79.1)	0.136
OXA-1^b^	52 (98.1)	34 (100.0)	0.105
OXA-9^b^	1 (1.9)	0 (0)	0.462
TEM-1D	25 (31.3)	31 (72.1)	<0.001
SHV-OKP-LEN^a^	2 (2.5)	43 (100.0)	<0.001
AmpC^a^	5 (6.3)	3 (7.0)	0.876
CMY	5 (100.0)	0 (0)	0.094
DHA	0 (0)	3 (100.0)	0.017
Male	38 (47.5)	20 (46.5)	0.917
Age, median [IQR]	78 [69.8–85]	79[67–84]	0.853
Charlson, median [IQR]	6[5–8]	7 [5–8.5]	0.482
APACHE II, median [IQR]	17 [11.8–20]	17 [11.5–24]	0.570
Pitt bacteremia score, me-dian [IQR]	1[0–2]	1[0–3]	0.431
ICU/HDU admission	5 (6.3)	5 (11.6)	0.298
Mortality	13 (16.3)	10 (23.3)	0.342
Empiric carbapenems	34 (42.5)	16 (37.2)	0.569
Definitive carbapenems	78 (97.5)	42 (97.6)	0.952
Community acquired	9 (11.3)	0 (0)	0.022
Nosocomial	21 (26.3)	30 (69.8)	<0.001
Healthcare associated	50 (62.5)	13 (30.2)	<0.001
Immunosuppressive state	16 (20.0)	13 (30.2)	0.202
Source of bacteremia:			
Urological	53 (66.3)	22 (51.2)	0.102
Respiratory	6 (7.5)	5 (11.6)	0.444
Hepatobiliary	9 (11.3)	3 (7.0)	0.446
Intraabdominal	5 (6.3)	3 (7.0)	0.876
Skin and soft tissue	2 (2.5)	1 (2.3)	0.952
Catheter related	1 (1.3)	4 (9.3)	0.031
Unknown	4 (5.0)	5 (11.6)	0.178

^a^Third-generation cephalosporin-resistant variants.

^b^Variants reported to hydrolyze broad-spectrum cephalosporins slightly^65^; OXA-1 confers resistance to piperacillin-tazobactam^66^, while OXA-9 has unknown effect on piperacillin-tazobactam.

Data are no. of patients (%), unless otherwise indicated.

IQR: Inter-quartile range; ICU: intensive care unit; HDU: high dependency unit.

Table 2 shows the 30-day mortality of empiric piperacillin-tazobactam and carbapenem groups and presence of beta-lactamase resistance mutations. Overall there was no significant difference in 30-day mortality between empiric piperacillin-tazobactam and carbapenem groups. The difference was also not significant by the type of beta-lactamase resistance mutations present.

**Table 2:** Beta-lactamases and 30-day mortality of empiric piperacillin-tazobactam and carbapenem groups.

Presence of beta-lactamase resistance genes	30-day mortality		p-value
	Empiric piperacillin-tazobactam (n = 73)	Empiric carbapenems (n = 50)	
CTX-M	11 (15.7)	10 (22.2)	0.378
OXA	9 (18.7)	11 (28.2)	0.297
TEM-1D	6 (15.8)	6 (33.3)	0.135
SHV-OKP-LEN	7 (25.0)	4 (23.5)	0.911
AmpC	0 (0)	3 (50.0)	0.206
Overall	11 (15.1)	12 (24.0)	0.212

Data are no. of patients who died (%).

**Figure 2:**
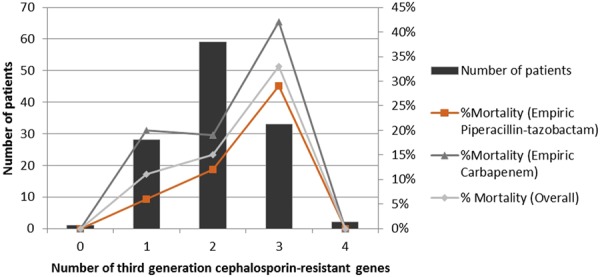
Correlation of 30-day mortality and the number of beta-lactamase genes which confer resistance to third-generation cephalosporins identified from *E. coli* and *K. pneumoniae* bacteremia.

The correlation between 30-day mortality and number of third-generation cephalosporin-resistant beta-lactamase genes detected in the infecting strains were investigated. When considering all SHV-OKP-LEN genes to be ESBL, overall, the number of resistance genes correlated with 30-day mortality (p = 0.045) as shown in Fig. 2; this correlation was found in the empiric piperacillin-tazobactam subgroup (p = 0.040) but not in the empiric carbapenem subgroup (p = 0.504). In both *E. coli* and *K. pneumoniae* subgroups, the correlation was not significant (E. coli: p = 0.073; K. pneumoniae: p = 0.433). However, multivariate analysis showed that the number of third-generation cephalosporin-resistant genes was not a significant risk factor for 30-day mortality (odds ratio [OR] 2.43, 95% confidence interval [CI]: 0.92–6.42) after adjusting for empiric carbapenem therapy, *K. pneumoniae* bacteremia, and Pitt bacteremia score ≥3.

Similar results were obtained when we repeated these analyses considering all SHV-OKP-LEN genes to be non-ESBL (i.e. p = 0.054 for correlation of resistance genes with 30-day mortality; no significant association in multivariate analysis [OR 2.43, p = 0.073]).

### Virulence gene analysis

An initial clustering showed that the *E. coli* and *K. pneumoniae* strains had different complements of virulence genes with clear clustering of strains by species; therefore, we analyzed the species separately.

For the 43 *K. pneumoniae* strains, a median of 60 virulence genes (range 27–81) was predicted in each strain; in total, 114 virulence genes were predicted in at least one of the *K. pneumoniae* strains. Of these 114 genes, 26 were found in all 43 strains; these were excluded from further analysis. Another 37 genes were predicted in only one of the 43 strains; these were also excluded. Among the remaining 51 virulence genes, the most prominent difference was the presence of genes for Yersiniabactin, an iron acquisition system^51^ present in 22/43 strains.

**Figure 3:**
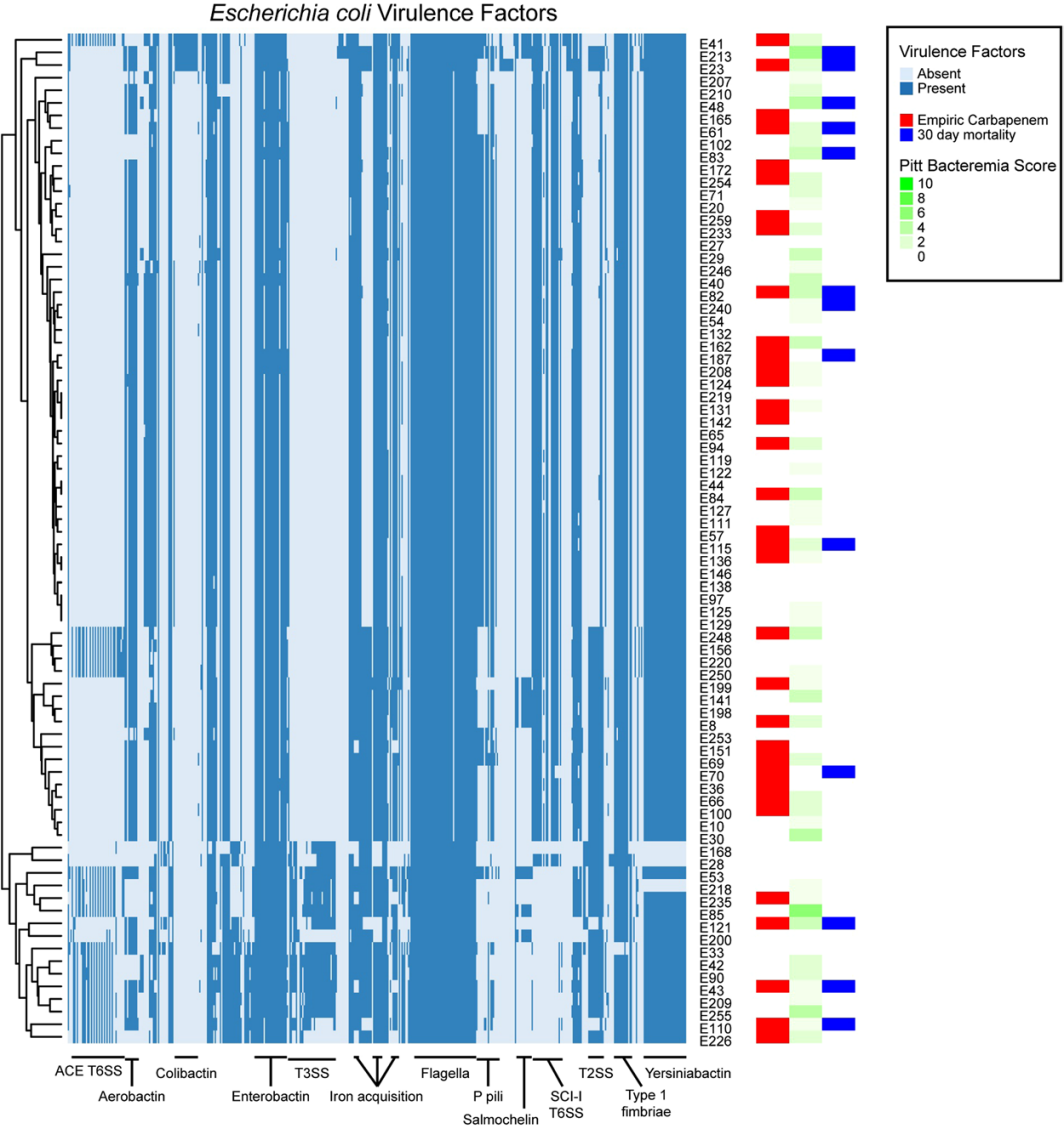
Heatmap of virulence factor calls from based on raw read mapping to VFDB for *K. pneumoniae* strains. Each row represents one *K. pneumoniae* strain, as indicated on the right of the heatmap. The strains are clustered based on their virulence factor profile, with the dendrogram displayed on the left. Each column represents one virulence factor, with selected general classes indicated below; a full list of virulence factors detected and their ordering in the heatmap can be found in Supplementary Table 1. Dark blue represents presence of a particular virulence factor in that strain; light blue indicates absence. Colored boxes on the right indicate empiric carbapenem, Pitt bacteremia score, and 30 day mortality associated with that strain.

*K. pneumoniae* strains with Yersiniabactin virulence gene seemed to have higher severity of illness and 30-day mortality. (Fig. 3) However, we tested all of the 51 variable *K. pneumoniae* virulence genes using Fisher’s exact test for significant association with empiric carbapenem treatment or with 30-day mortality; none of these was significantly associated after a Bonferroni correction for multiple testing.

**Figure 4:**
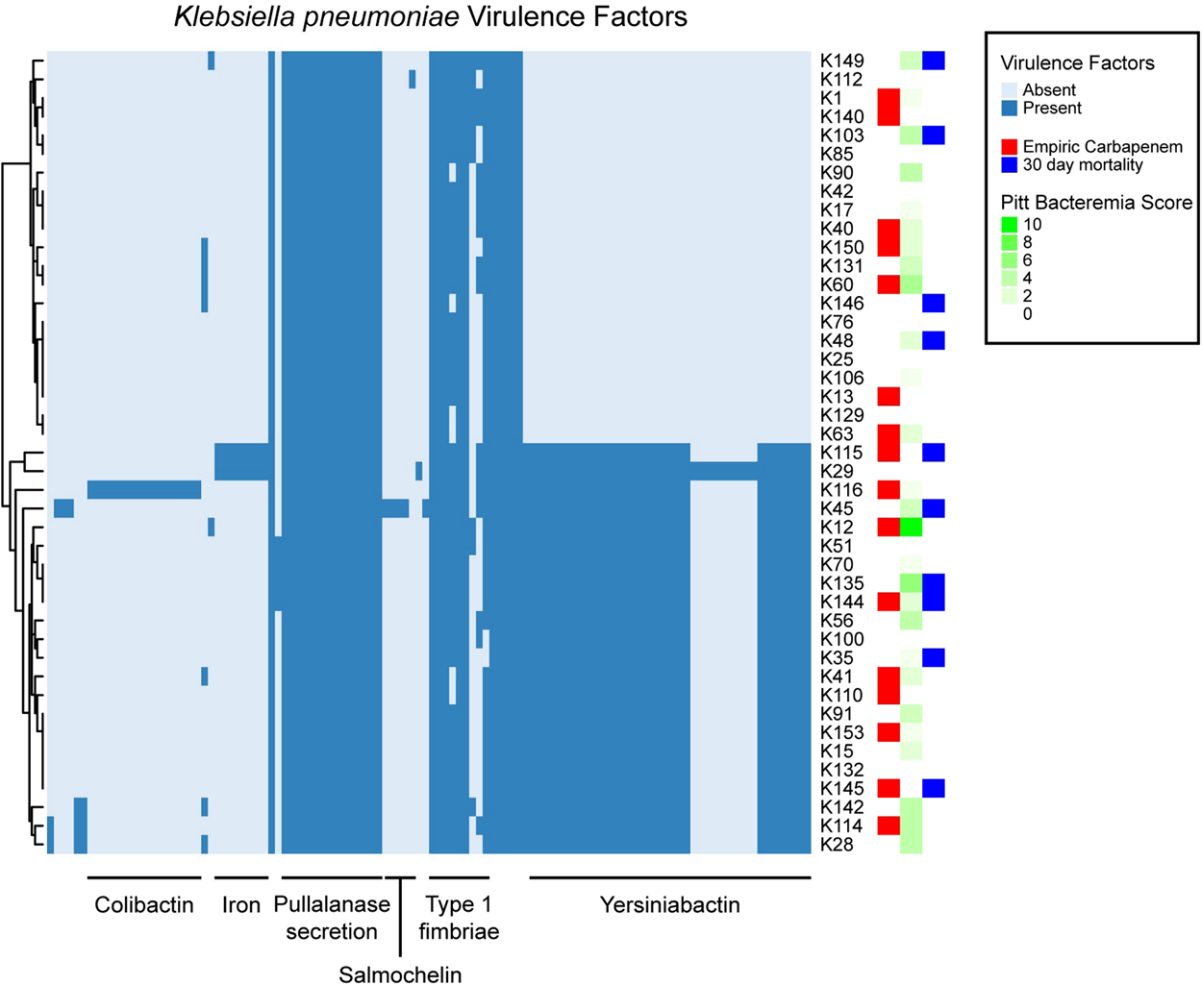
Heatmap of virulence factor calls from based on raw read mapping to VFDB for *E. coli* strains. Each row represents one *E. coli* strain, as indicated on the right of the heatmap. The strains are clustered based on their virulence factor profile, with the dendrogram displayed on the left. Each column represents one virulence factor, with selected general classes indicated below; a full list of virulence factors detected and their ordering in the heatmap can be found in Supplementary Table 1. Dark blue represents presence of a particular virulence factor in that strain; light blue indicates absence. Colored boxes on the right indicate empiric carbapenem, Pitt bacteremia score, and 30 day mortality associated with that strain.

For the 80 *E. coli* strains, a median of 237 virulence genes (range 164–304) was predicted in each strain; in total, 480 genes were predicted in at least one strain, 105 were present in 79 or 80 strains, and 39 were present in exactly one strain; these were excluded from further analysis. Major differences among the strains included genes encoding the ACE and SCI-I Type VI secretion systems, the ETT2 Type III secretion system, and a Type II secretion system, as well as P fimbriae and the general secretion pathway. In general, strains carried genes similar to either the ACE or the SCI-I Type VI secretion system (Fig. 4); those with the SCI-I Type VI system tended to carry P pili and to lack the ETT2 Type III system. The Type II secretion system and the general secretion pathway tended to co-occur in strains and were split among those strains carrying the ACE or the SCI-I Type VI secretion systems. The ST131 strains were those that tended to carry the SCI-I Type VI secretion system and to not carry the ETT2 Type III secretion system. We tested the association between the 336 variable genes and empiric carbapenem treatment or 30-day mortality. Again, none of these virulence factors was significantly associated with either of these clinical variables. We also tested for an association of these clinical variables with ST131; neither of these was significant. Finally, we tested for an association using only variable genes among ST131 or non-ST131 strains separately; again, none of these tests was significant. No statistically significant correlation was found between virulence factors and Pitt bacteremia score after Bonferroni p-value correction.

## Discussion

We initially found a significant correlation between increased 30-day mortality and the number of beta-lactamase genes that confer third-generation cephalosporin resistance. The correlation seemed to be present when piperacillin-tazobactam was used but not with carbapenem, suggesting that carbapenems may be more stable in presence of multiple broad spectrum beta-lactamases. However, after adjusting for empiric carbapenem therapy, *K. pneumoniae* bacteremia and Pitt bacteremia score 3, no significant association was found between the number of resistant genes and mortality. None of the individual beta-lactamase genes were associated with mortality according to the type of ESBL’s consistent with the findings from other studies^52–54^. Mortality rate seemed to be lower in the piperacillin-tazobactam group compared with the carbapenem group; propensity analysis in our previous publication revealed carbapenems were more likely to be used for treatment in sicker patients, explaining higher mortality in that group^28^.

In our multivariate analysis on 30-day mortality, we aimed to explore the effect of microbiological determinants on patient outcome in presence of clinical variables. High Pitt bacteremia score was found to be a significant risk factor for mortality (OR 3.53, 95% CI: 1.23–10.13), similar to findings in other studies^27,30,31^. Comorbidities were not significantly associated with patient mortality in the univariate analysis (OR 1.14, 95% CI: 0.96–1.36), and therefore was excluded from multivariate analysis. We did not look at the effect of definitive therapy (defined as antimicrobial therapy that was continued or commenced on the day that the antibiotic susceptibility results were reported to the clinicians) on mortality as most of the patients received carbapenems for definitive therapy (120/123 [98%]).

Tazobactam was reported to be active against TEM ESBL but not SHV ESBL^6^ although a high bacterial inoculum could confer resistance to tazobactam^55^. There was certainly a high prevalence of TEM and SHV in our study but we did not find that their presence affected 30-day mortality. In this study, SHV were included with OKP and LEN from the ARGAnnot database as they are relatively closely related by DNA sequence. We performed gene analyses similarly to other recent publications on determining resistance genes for ESBL organisms^56,57^. Among the strains positive for SHV-OKP-LEN, all predicted genes were SHV alleles. SHV-1, a commonly reported non-ESBL variant, was not found in any of our strains, although the SHV variants in our study were not all ESBL variants. We found that the ESBL classification of SHV-OKP-LEN genes did not significantly affect the correlations with mortality (p = 0.054 when SHV-OKP-LEN was omitted in correlation analyses) and multivariate analysis (OR 2.43, p = 0.073).

Tazobactam was found to be 100 time more active against CTX-M than clavulanate^4^, and CTX-M is the predominant ESBL in our study. However, multiple ESBL’s, hyper-production of non-ESBL beta-lactamases like TEM and SHV, and beta-lactamase production with porin loss may reduce the effectiveness of piperacillin-tazobactam^4^. This may explain our observation of significant association of number of resistant genes with 30-day mortality in the piperacillin-tazobactam group, which was lost after adjustment for confounders. Piperacillin-tazobactam was not considered active against AmpC beta-lactamases *in vitro*^11^, which was not very common in our study, although a meta-analysis of clinical studies did not find piperacillin-tazobactam was inferior to carbapenems in treating AmpC-producing gram negative bacteremia^58^.

As the *E. coli* and *K. pneumoniae* strains in our data set had very different virulence gene complements, we did not perform a combined analysis of virulence factors and mortality. This is unlike that of beta-lactamase resistance genes as both *E. coli* and *K. pneumoniae* were found to harbor similar beta-lactamases. There was no clear association between virulence factors and 30-day mortality in either *E. coli* or *K. pneumoniae* groups. This is in contrast with the findings from 3 clinical studies on *E. coli*, which found association between certain virulence factors and mortality; particularly *papGII* and hemolysin production were reported to have a protective effect on mortality^52,59,60^, whereas *ibeA* (which promotes invasion)^52^ and *fyuA* (encoding for Yersiniabactin siderophore receptor)^59^ increased mortality. Of note, among our *E. coli* strains, at least one hemolysin gene was found in all 80 strains, and 77 strains carried the *fyuA* gene and most of the genes annotated as required for Yersiniabactin production (all death cases were among these 77). Only two strains carried the *ibeA* gene; neither was from a death case. Forty five *E. coli* strains carried the *papG* gene, of which 44 were *papGII* and 1 was *papGIII*; 6 of 13 death cases were associated with strains carrying *papGII*. Thus none of these previously reported *E. coli* virulence genes were significantly associated with mortality in our data set. The role of virulence factors on mortality is still unclear in other studies^60–62^, although virulence factors and resistance genes were reported to affect each other in various ways. Specifically, a higher proportion of ESBL-producing *K. pneumoniae* was found to produce both type 1 and 3 fimbrial adhesins than non-ESBL strains, whereas *E. coli* with OXA-10 like and SFO-1 beta-lactamases were found to have a statistically significant fitness cost^63^. A study by Lefort *et al*. suggested that host factors and portal of entry outweighed bacterial determinants on mortality of patients with *E. coli* bacteremia^64^. In our multivariate analysis, only high Pitt bacteremia score was associated with mortality, suggesting that bacterial determinants may not be a significant influence when active piperacillin-tazobactam or carbapenems are given empirically.

Our study has several limitations. It was a retrospective analysis and we may not be able to control for all possible confounders. We retrieved only 124 isolates from our microbiology department out of the 151 patients included in the previous study^28^, and therefore we could not analyze the resistance mutations of the remaining 27 patients. We believe however that this will not affect the validity of our results. Our study sample size was low overall, so some subgroups (e.g. AmpC) likely did not have enough power to detect associations. At our current available sample size, the power to detect the same difference is 0.73. To detect a 20% difference in mortality between patients treated with piperacillin-tazobactam and carbapenem with 80% power, a sample of 160 patients would be required. Therefore, the primary value of this study in looking at effects of type of antibiotics, resistance genes, and virulence factors on mortality remains exploratory. We did not evaluate the minimum inhibitory concentration of empiric antibiotic used as it was not routinely tested in all cases, which could be a significant factor on 30-day mortality. Lastly, this study only included *E. coli* and *K. pneumoniae* samples with ESBL and/or AmpC beta-lactamases and may not be generalizable to patients with other types of infections. We did not examine the presence of efflux pumps or porin loss which may modify the efficacy of both piperacillin-tazobactam and carbapenems.

## Conclusion

In conclusion, we did not find significant association between 30-day mortality and any ESBL and AmpC beta-lactamases, as well as any virulence factors when patients were given either piperacillin-tazobactam or carbapenems empirically. New studies in this area are much needed as clinical trials are underway to investigate effectiveness of piperacillin-tazobactam for treatment of ESBL-producing gram negative bacteremia^33^.

## Electronic supplementary material

Supplementary data

## Data Availability

All data generated or analysed during this study are included in this published article (and its Supplementary Information files).
