# Supplementary material for: No association between resistance mutations, empiric antibiotic, and mortality in ceftriaxone-resistant Escherichia coli and Klebsiella pneumoniae bacteremia
Source: Sci Rep. 2018 Aug 24;8:12785. doi: 10.1038/s41598-018-31081-6 (PMC6109088; doi:10.1038/s41598-018-31081-6)
Supplement: Supplementary file 1 — Supplementary data [file 41598_2018_31081_MOESM1_ESM.pdf]

Supplementary Data
No association between resistance mutations, empiric antibiotic, and mortality in ceftriaxone-resistant *Escherichia coli* and *Klebsiella pneumoniae* bacteremia

Shi Thong Heng[1], Swaine L. Chen[2,3], Joshua G.X. Wong[4], David C. Lye[3,4,5], and Tat Ming Ng[1]

[1]Department of Pharmacy, Tan Tock Seng Hospital, Singapore
[2]GERMS and Infectious Diseases Group, Genome Institute of Singapore, Singapore
[3]Department of Medicine, Yong Loo Lin School of Medicine, National University of Singapore, Singapore
[4]Department of Infectious Diseases, Tan Tock Seng Hospital, Singapore
[5]Lee Kong Chian School of Medicine, Nanyang Technological University, Singapore

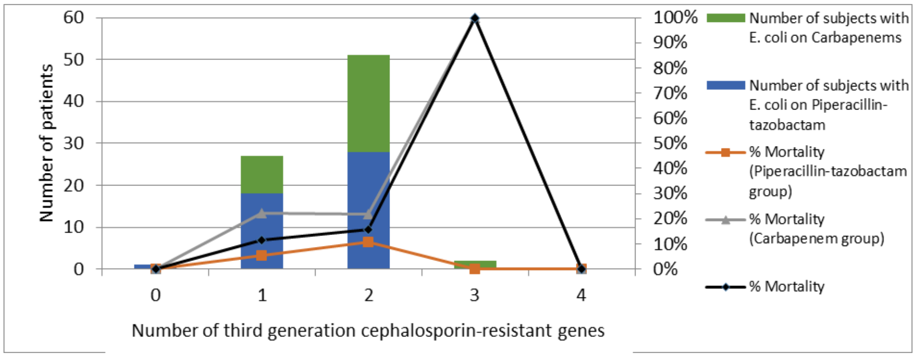

Figure S1. Correlation of 30-day mortality and the number of beta-lactamase genes with third-generation cephalosporin resistance in patients with *E. coli* bacteremia (p=0.021, Mantel-Haenszel linear by linear association test)

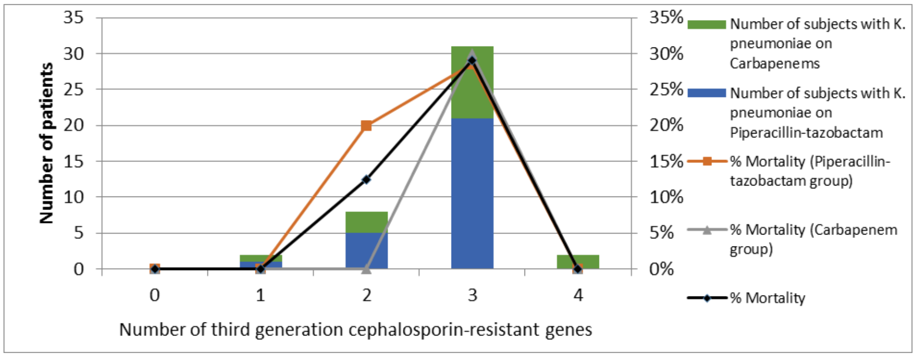

Figure S2. Correlation of 30-day mortality and the number of beta-lactamase genes with third-generation cephalosporin resistance in patients with *K. pneumoniae* bacteremia (p=0.793, Mantel-Haenszel linear by linear association test)

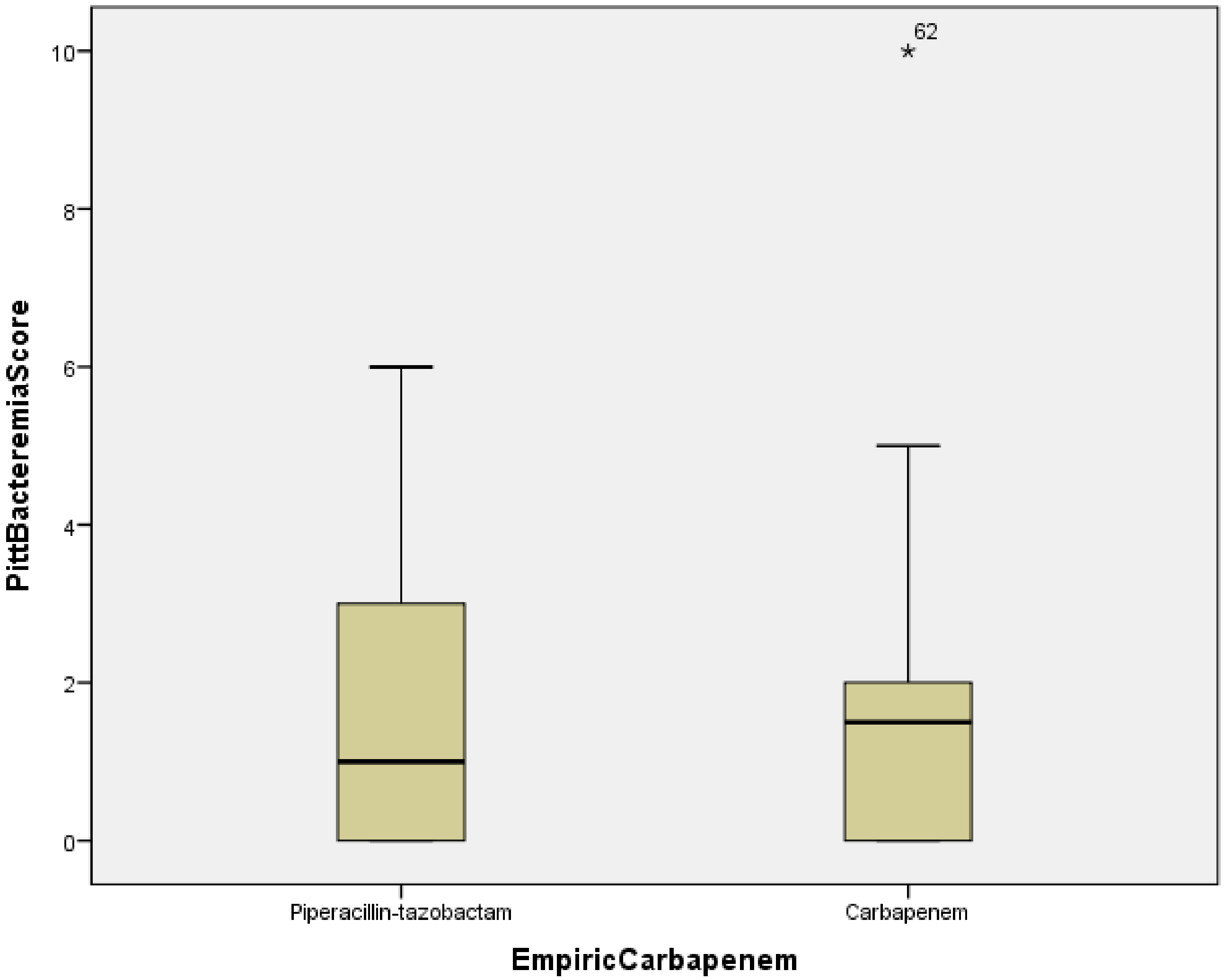

Figure S3. Box plot of Pitt Bacteremia Score in empiric piperacillin-tazobactam and carbapenem groups)

Presence of beta-lactamase resistance mutations	30-day Mortality		p-value
	Empiric Piperacillin-tazobactam (n=46)	Empiric Carbapenems (n=34)	
CTX-M	4 (9.9)	7 (21.9)	0.118
OXA	3 (11.1)	8 (30.8)	0.078
TEM-1D	1 (6.3)	3 (33.3)	0.076
SHV-OKP-LEN	0 (0)	1 (100.0)	0.157
AmpC	0 (0)	3 (100.0)	0.025
**Overall**	4 (9%)	9 (26%)	0.033

Data are no. of patients who died (%), unless otherwise indicated

Table S1. Beta-lactamases and mortality rate of empiric piperacillin-tazobactam and carbapenem groups in *E. coli* bacteremia

Presence of beta-lactamase resistance mutations	30-day Mortality		p-value
	Empiric Piperacillin-tazobactam (n=27)	Empiric Carbapenems (n=16)	
CTX-M	7 (26.9)	3 (23.1)	0.795
OXA	6 (28.6)	3 (23.1)	0.724
TEM-1D	5 (22.7)	3 (33.3)	0.540
SHV-OKP-LEN	7 (25.9)	3 (18.8)	0.590
AmpC	0 (0)	0 (0)	-
**Overall**	7 (26%)	3 (19%)	0.590

Data are no. of patients who died (%), unless otherwise indicated

Table S2. Beta-lactamases and mortality rate of empiric piperacillin-tazobactam and carbapenem groups in *K. pneumoniae* bacteremia

Independent factors	OR	95% CI	p-value
Empiric carbapenem	1.780	0.715-4.432	0.215
K. pneumoniae	1.562	0.620-3.934	0.344
Pitt bacteremia score>=3	2.929	1.099-7.807	0.032
Number of resistance mutations with third-generation cepahlosporin-resistant variants	1.873	1.005-3.490	0.048
OXA	3.284	0.910-11.846	0.069
AmpC	2.850	0.629-12.906	0.174
CTX-M	0.670	0.126-3.556	0.638
TEM-1D	1.388	0.560-3.444	0.479
SHV-OKP-LEN	1.779	0.711-4.451	0.218
Male	0.420	0.159-1.110	0.080
Age	1.018	0.977-1.061	0.396
Charlson score	1.139	0.956-1.356	0.146
APACHE II score	1.091	1.023-1.164	0.008
Community acquired infection	1.265	0.245-6.531	0.779
Nosocomial infection	1.107	0.443-2.766	0.828
Healthcare associated infection	0.846	0.342-2.097	0.718
Intraabdominal	0.604	0.071-5.165	0.645
Respiratory	6.706	1.838-24.463	0.004
Unknown source	1.265	0.245-6.531	0.779
Urological	0.995	0.393-2.518	0.991
Immunosuppresive state	0.880	0.295-2.621	0.818

OR: Odds ratio, CI: confidence interval

Table S3. Univariate analysis of 30-day mortality for 123 patients with *E. coli* and *K. pneumoniae* bacteremia, receiving either empiric carbapenem or piperacillin-tazobactam.

Independent factors	OR	95% CI	p-value
Empiric carbapenem	1.873	0.696-5.041	0.214
K. pneumoniae	0.541	0.126-2.320	0.409
Pitt bacteremia score>=3	3.529	1.229-10.133	0.019
Number of resistance mutations with third-generation cepahlosporin-resistant variants	2.430	0.920-6.422	0.073

OR: Odds ratio, CI: confidence interval
Hosmer and Lemeshow test p value=0.417

Table S4. Multivariate analysis of 30-day mortality for 123 patients with *E. coli* and *K. pneumoniae* bacteremia, receiving either empiric carbapenem or piperacillin-tazobactam.
